# Shorter time-to-positivity and turnaround time with mycosis blood culture bottles when detecting *Candida albicans*

**DOI:** 10.1007/s15010-024-02216-x

**Published:** 2024-02-23

**Authors:** Jacqueline Färber, Achim J. Kaasch, Enrico Schalk

**Affiliations:** 1 Medical Faculty, Institute of Medical Microbiology and Hospital Hygiene, Otto von Guericke University Magdeburg, Magdeburg, Germany; 2 Department of Hematology, Oncology and Cell Therapy, Medical Faculty, Otto von Guericke University Magdeburg, Leipziger Str. 44, 39120 Magdeburg, Germany

*Candida* spp. are human commensals and are commonly found on skin and throughout the gastrointestinal tract. Central venous catheters (CVC) and an immunocompromised state are major risk factors for candidemia [1, 2]. Thus, bloodstream infections (BSI) due to *Candida* spp. are more common in patients with hematological malignancies (1.4 vs. 0.83 cases/1,000 admissions) [2]. *Candida* BSI is associated with a higher 28-day all-cause mortality in hospitalized patients with hematological malignancies (45 vs. 11%) [2]. Early diagnosis, rapid initiation of appropriate treatment, and prompt source control (e.g. CVC removal) is key for reducing mortality in candidemia [3, 4].

Blood cultures (BC) are the gold standard to diagnose BSI, including candidemia [5]. The time-to-result, or turnaround time (TAT), for the microscopic result, in BC diagnostics depends on several factors: First, the incubation delay (“time-to-machine”), i.e. the time needed until the BC bottle is inoculated, transported to the BC instrument, and incubation is started. Second, the “time-to-positivity” (TTP), i.e. the time needed by the BC instrument to detect microorganisms in the BC bottle. Third, the “result-to-report” time, i.e. the time needed for processing and reporting a Gram stain. The incubation delay and the result-to-report time are heavily dependent on organizational processes like transport and laboratory working hours.

Aerobic and anaerobic BC media are not optimal for the growth of all yeast species [5]. This can be exploited for predicting the presence of *C. glabrata* with high specificity when earlier detection of growth occurs in anaerobic BC media [6]. However, false-negative BC results are life-threatening, especially in high-risk, neutropenic patients. About 50% of BC in candidemia are false-negative, but BC positivity can be increased by culturing a higher volume of blood (i.e. two aerobic bottles and one anaerobic BC bottle), or by using a broth medium designed for an enhanced yield of yeasts (so-called fungus or mycosis BC) [5].

So far, guidelines do not recommend a higher blood volume or mycosis BC for hematology patients [7, 8], or patients with CVC [9, 10], who are at higher risk for candidemia.

In this study, we aimed to evaluate the influence of different BC media and incubation delays on the ability to detect yeasts. In brief, blood from healthy donors was warmed up to 38.3 °C, mimicking a febrile patient [8] with hematogenous dissemination of yeasts due to e.g. chemotherapy-induced mucositis, graft-*vs.*-host disease, or an indwelling CVC. Then, the blood was immediately inoculated with *C. albicans,* the most common causative species found in candidemia [1]. The prepared blood sample was transferred into BACTEC™ Plus Aerobic/F (“Aerobic”), Anaerobic/F (“Anaerobic”), Lytic 10 Anaerobic/F (“Lytic”), and Mycosis-IC/F (“Mycosis”) BC bottles and incubated in a BC instrument immediately (hour 0), or stored at 20–25 °C and transferred with a delay of 2, 4, 8, 12, and 16 h, respectively. BC bottles were incubated until positivity or for a maximum of 5 days. All BC were sub-cultured to verify the growth in every bottle. Each experiment was repeated three times for each of the six different time points. In a simulated scenario, the TAT was calculated considering an incubation delay due to transport times of 2, 4, 8, 12, and 16 h as well as laboratory working hours from 07:00 a.m. to 6:00 p.m. on workdays with the TTP data form this experiment. Details for materials and methods are described in the Supplementary Material.

In total, 72 BC were spiked with *C. albicans*. None of the 18 Lytic BC bottles became positive, however, growth was detected in 5/18 (27.8%) in subcultures, indicating that yeasts are viable in the lytic medium for several days. Only 7/18 (38.9%) Anaerobic BC turned positive. However, in all 11 subcultures with no positive signal growth was possible (100%).

In all 18 Mycosis BC as well as in all 18 Aerobic BC a positive signal was detected. We found a shorter TTP for Mycosis BC compared to Aerobic BC for all time points; with a mean 13.71 h (23.22 vs. 36.93 h; *p* < 0.001) shorter TTP over all time points (Table 1).

**Table 1:** Time-to-positivity for Mycosis and Aerobic blood cultures

Incubation delay, h	Mycosis BC TTP^a^ [h]	Aerobic BC TTP^a^ [h]	Difference TTP^a^ [h]	*p* value
0	25.42 ± 1.11	40.53 ± 5.13	15.11	0.008
2	34.57 ± 17.02	53.39 ± 12.06	18.81	0.19
4	24.16 ± 0.97	38.78 ± 2.84	14.62	0.001
8	22.31 ± 0.35	30.93 ± 0.34	8.63	< 0.001
12	22.06 ± 1.04	34.77 ± 6.19	12.71	0.025
16	18.11 ± 0.58	23.16 ± 0.91	5.05	0.001

Times are stated as means

*BC* blood culture, *TTP* time-to-positivity

^a^ ± standard deviation

In a simulated scenario taking into account the transport time and the laboratory working hours, the mean TAT for Mycosis BC were significant shorter compared to Aerobic BC (*p* < 0.001 for all), with a difference up to 24 h (Table S1).

Furthermore, we analyzed data from 948 BC drawn in clinical routine from 08/02/2020 to 07/28/2021 in the Department of Hematology and Oncology. Roughly half (463/948, 48.8%) of all BC were drawn in the late afternoon and evening, i.e. between 4:00 p.m. and 10:00 p.m. Only 62 of these 463 (13.4%) BC arrived at the laboratory on the same day; 320 (69.9%) BC arrived the next day in the morning hours (between 7:00 a.m. and 10:00 a.m.). Blood cultures received in the morning hours had a median transport time of 10.0 h (range 1–23).

As in other institutions [11], the “time to machine” shows potential for improvement, with a TAT of 1.5 days for a microscopic result. Although, an incubation delay results in a shorter TTP [11], our analysis shows, that this cannot compensate for an increased TAT. Key drivers for a longer TAT were the reduced availability of transportation and laboratory workforce during evening and nighttime. This could be partially compensated for by satellite BC systems, however, their clinical utility remains to be determined [12].

Our results show that the detection of *C. albicans* can be markedly improved by using Mycosis BC bottles. However, previous antifungal therapy, especially with echinocandins, was reported to increase TTP in Mycosis BC bottles [13, 14]. Further, our results cannot be extrapolated to other fungal species and other BC systems. For example, the recovery rate for *C. glabrata* was significantly lower in the BACTEC™ than in the BacT/ALERT^®^ system, and it is recommended to include the mycosis medium in BC at centers that use the BACTEC™ system and for patients at risk for candidemia [15]. Given our results, we propose to incorporate the use of specialized BC media into relevant guidelines for patients without previous antifungal treatment with a high risk for candidemia, e.g. in hematology. This may result in earlier treatment and source control with improved prognosis.

### Supplementary Information

Below is the link to the electronic supplementary material.

Supplementary file1 (DOCX 134 kb)

## Data Availability

The data that support the findings of this study are available from the corresponding author upon reasonable request.
