# Supplementary material for: Shorter time-to-positivity and turnaround time with mycosis blood culture bottles when detecting Candida albicans
Source: Infection. 2024 Feb 23;52(2):701–3. doi: 10.1007/s15010-024-02216-x (PMC10955001; doi:10.1007/s15010-024-02216-x)
Supplement: Supplementary file 1 — Supplementary file1 (DOCX 134 kb) [file 15010_2024_2216_MOESM1_ESM.docx]

***Supplementary Material***

**Shorter time-to-positivity and turnaround time with mycosis blood culture bottles when detecting *Candida albicans***

Jacqueline Färber^1^, Achim J Kaasch^1^, Enrico Schalk^2^

^1^Institute of Medical Microbiology and Hospital Hygiene, Medical Faculty, Otto von Guericke University Magdeburg, Magdeburg, Germany

^2^Department of Hematology, Oncology and Cell Therapy, Medical Faculty, Otto von Guericke University Magdeburg, Magdeburg, Germany

**Material and Methods**

The study protocol (ENTRY, Effects of delayed Entry into Blood Culture Systems on Culture Positivity) was approved by the Ethics Committee of the Medical Faculty of the Otto von Guericke University Magdeburg, Magdeburg, Germany (approval no. 115/14). All blood donors gave written informed consent.

In the ENTRY study, a maximum of 378 mL whole blood from healthy donors was drawn in 7 mL Na-Heparin vacutainers (*n*=54) and was stored up to three days at 4°C. For spiking blood with yeasts, one vacutainer per experiment with 7 mL of individual blood was warmed up to 38.3°C and immediately inoculated with *Candia albicans* ATCC 14035 in saline suspension at a concentration of 1 to ≤5 CFU/mL. The inoculum was prepared as follows: *C. albicans* was cultured from frozen stock with two consecutive subcultures on 5% Sheep Blood Agar plates for 16-24 hours in an aerobic atmosphere at 35 ± 1°C. Colonies were collected from the second subculture and transferred into a sterile solution of 0.9% sodium chloride. Subsequently, serial dilution was performed and the concentration of viable yeasts in suspension was determined by culture on 5% Sheep Blood Agar plates.

Spiked 7 mL blood was transferred in BACTEC^™^ Plus Aerobic/F (“Aerobic”), Anaerobic/F (“Anaerobic”), Lytic 10 Anaerobic/F (“Lytic”) and Mycosis-IC/F (“Mycosis”) blood culture (BC) bottles and incubated in BACTEC^™^ 9120 or 9240 BC instruments. The inoculated BC bottles were transferred into the incubator immediately (hour 0) or stored at 20-25°C and transferred with a delay of 2, 4, 8, 12, and 16 hours, respectively, for a 5-day incubation protocol. Upon detection of one or more positive BC bottles, the time-to-positivity (TTP) was noted. After the incubation period, all bottles (positive signal and no positive signal) were sub-cultured with two drops from each bottle on standard bacteriology media at 35 ± 1°C in an aerobic atmosphere to verify the growth in every bottle. Each experiment was repeated three times for each of the six different time points. A summary and example is given in **Fig. S1**.

All materials and equipment mentioned here were by Becton Dickinson, Heidelberg, Germany.

We addressed the influence of incubation delay due to transport and opening hours on the turnaround time (TAT) by simulation. TAT was calculated summing up the incubation delay (transport times of the BC of 2, 4, 8, 12, and 16 hours), delays caused by the laboratory working hours (from 07:00 a.m. to 6:00 p.m. on workdays), and the TTP data derived from this study (an example is given in **Fig. S2**: a BC drawn at 03:00 a.m., transport time of four hours, the BC instrument signals for positivity after 38 hours, reporting delay of ten hours – in sum, the TAT was 52 hours).

**Statistical analyses**

For comparison of means of TTP and TAT Student’s t-test was used. Two-sided *p* values <0.05 were considered statistically significant. Statistical analysis was carried out using SPSS, version 26 (IBM, Armonk, NY, USA).

**Supplementary Figures**


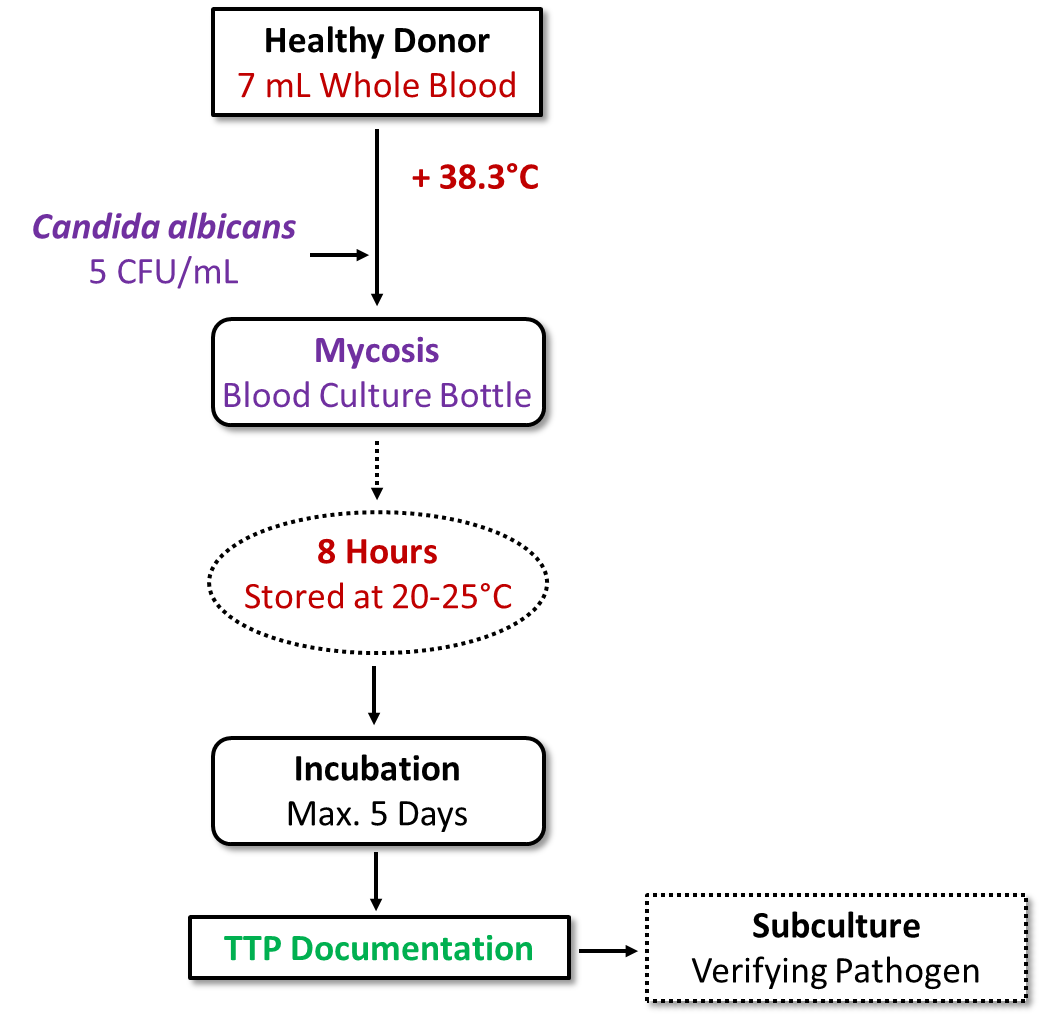


**Supplementary Fig. S1** Flow chart of the experiment, example for Mycosis blood culture bottle, incubation delay of 8 hours.

TTP, time-to-positivity.


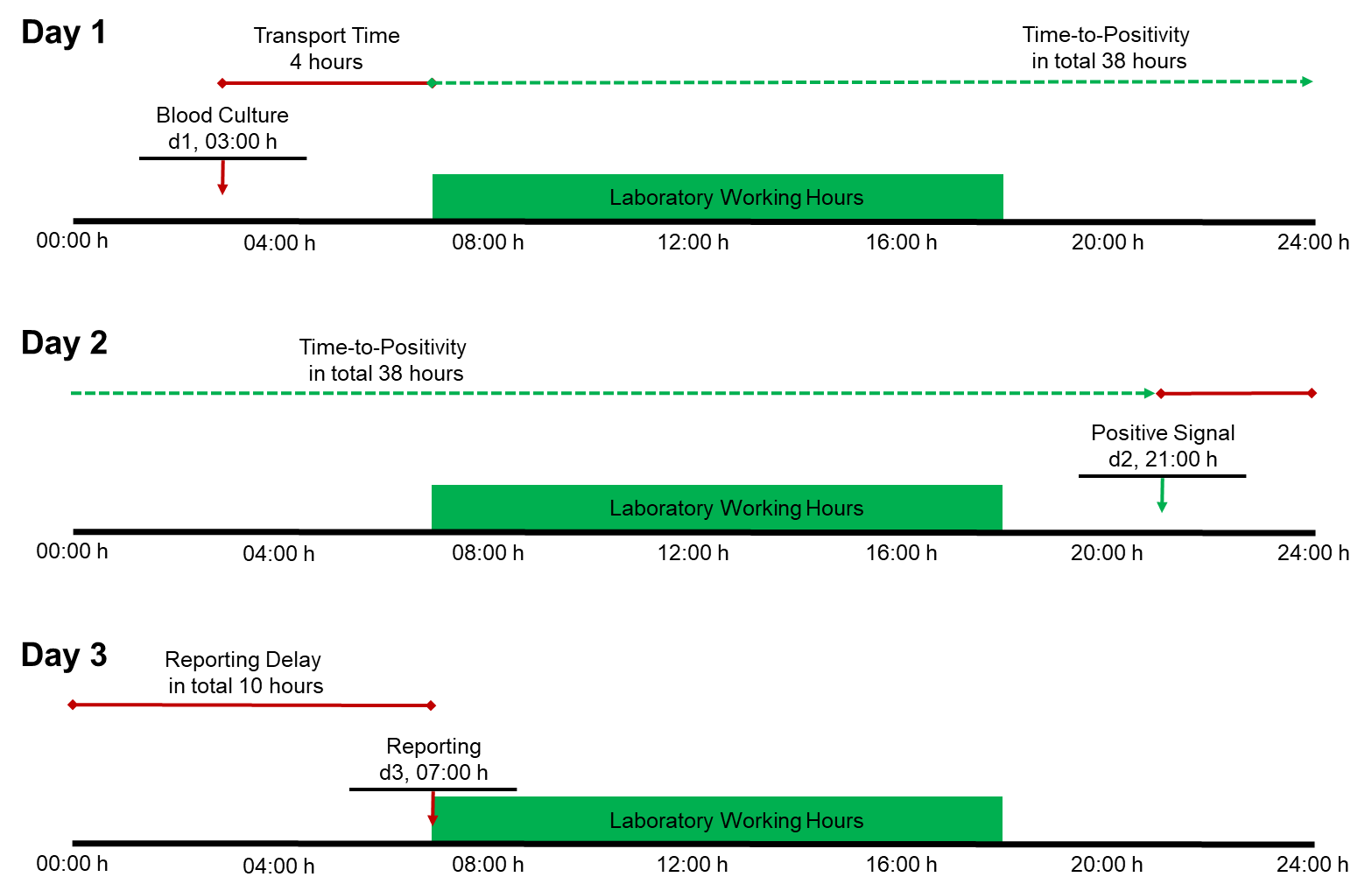


**Supplementary Fig. S2** Example for determination of turnaround time.

**Supplementary Table**

| **Table S1.** Simulated turnaround time for Mycosis and Aerobic blood cultures | | | | |
| --- | --- | --- | --- | --- |
| **Incubation delay**  **Hour** | **Mycosis BC TAT [h]**  **(range^b^)** | **Aerobic BC TAT [h]**  **(range^b^)** | **Difference TAT [h]**  **(range^b^)** | ***P* value** |
| 2 | 42.25  (36 – 48) | 59.25  (55 – 67) | 17.00  (7 – 24) | <.001 |
| 4 | 27.75  (28 – 28) | 46.58  (42 – 52) | 18.83  (14 – 24) | <.001 |
| 8 | 30.23  (30 – 32) | 41.92  (38 – 49) | 11.69  (6 – 19) | <.001 |
| 12 | 34.08  (34 – 35) | 51.00  (46 – 57) | 16.92  (11 – 23) | <.001 |
| 16 | 35.62  (34 – 40) | 39.08  (39 – 40) | 3.46  (0 – 5) | <.001 |

**Note.** Times are stated as means.

BC, blood culture; TAT, turnaround time.

^a^Rounded down to whole hours.
